# Unique Genomic Alterations of Cerebrospinal Fluid Cell-Free DNA Are Critical for Targeted Therapy of Non-Small Cell Lung Cancer With Leptomeningeal Metastasis

**DOI:** 10.3389/fonc.2021.701171

**Published:** 2021-10-04

**Authors:** Yongsheng Wang, Feng Jiang, Ruixue Xia, Ming Li, Chengyun Yao, Yan Li, Hui Li, Qi Zhao, Mingke Shi, Yanzhe Yu, Yang W. Shao, Guoren Zhou, Hongping Xia, Liyun Miao, Hourong Cai

**Affiliations:** ^1^ Department of Respiratory Medicine & Radiology & Cardiothoracic Surgery, Nanjing Drum Tower Hospital Affiliated to Medical School of Nanjing University, Nanjing, China; ^2^ The First Affiliated Hospital/Yijishan Hospital of Wannan Medical College, Wuhu, China; ^3^ Department of Respiratory and Critical Care Medicine, Henan University Huaihe Hospital, Kaifeng, China; ^4^ Jiangsu Cancer Hospital, The Affiliated Cancer Hospital of Nanjing Medical University, Jiangsu Institute of Cancer Research, Nanjing, China; ^5^ Nanjing Geneseeq Technology Inc., Nanjing, China; ^6^ Department of Pathology, School of Basic Medical Sciences & Sir Run Run Hospital & Key Laboratory of Antibody Technique of National Health Commission, Nanjing Medical University, Nanjing, China

**Keywords:** non-small cell lung cancer, leptomeningeal metastasis, cerebrospinal fluid, liquid biopsy, cell-free DNA

## Abstract

We reported unique molecular features of cerebrospinal fluid (CSF) of nonsmall cell lung cancer (NSCLC) patients with leptomeningeal metastasis (LM), suggesting establishing CSF as a better liquid biopsy in clinical practices. We performed next-generation panel sequencing of primary tumor tissue, plasma, and CSF from 131 NSCLC patients with LM and observed high somatic copy number variations (CNV) in CSF of NSCLC patients with LM. The status of EGFR-activating mutations was highly concordant between CSF, plasma, and primary tumors. ALK translocation was detected in 8.3% of tumor tissues but only 2.4% in CSF and 2.7% in plasma. Others such as ROS1 rearrangement, RET fusion, HER2 mutation, NTRK1 fusion, and BRAF V600E mutation were detected in 7.9% of CSF and 11.1% of tumor tissues but only 4% in plasma. Our study has shed light on the unique genomic variations of CSF and demonstrated that CSF might represent better liquid biopsy for NSCLC patients with LM.

## Introduction

The incidence of leptomeningeal metastasis (LM) of nonsmall cell lung cancer (NSCLC) is increasing due to improved treatment and ultimately prolonged survival of NSCLC patients (1, 2). However, the survival of NSCLC with LM is still poor, with the overall survival varying from 3 to 11 months after diagnosis (3–5). The poor survival is largely due to the blood-brain barrier, which restricts the transport of therapeutic agents and creates a so-called sanctuary site for tumor cells (6, 7). The molecular mechanism of LM is of critical importance to be understood, especially the genetic characteristics of the tumor cells that can invade the leptomeningeal space.

The individual targeted therapy based on molecular genotyping has increasingly improved the survival of NSCLC (8–10). Epidermal growth factor receptor (EGFR) tyrosine kinase inhibitors, for example, have increased the survival of patients with EGFR mutation (11, 12). Anaplastic lymphoma kinase (ALK) inhibitor has also extended the survival of NSCLC patients with ALK translocation (13). Other actionable targets such as c-ros oncogene 1 (ROS1), BRAF V600E, and neurotrophic tyrosine receptor kinase (NTRK) also have tyrosine kinase inhibitors other than chemotherapy alone (14–16). Tissue biopsy was regarded as the golden standard of molecular classification and was critical in decision-making concerning treatment for advanced NSCLC patients (17). However, it is a clinical challenge to collect tumor tissue for genotyping, as the invasive and time-consuming procedures may be risky to these advanced-stage NSCLC patients (18). Liquid biopsy, based on body fluids including plasma, urine, and other liquids, including CSF, can detect tumor-related biomarkers to predict clinical outcomes safely and early in the management of NSCLC (19). Previous studies indicated that CSF could be an important liquid biopsy method in patients with central nervous system (CNS) cancers. However, for NSCLC patients with LM, a small number of patients has been studied and the genotyping of CSF for NSCLC with LM has been largely unknown (2, 20).

Our current study, revealing the genetic alterations of CSF and comparing the genetic difference of CSF with matched plasma and tissue samples, provides stronger evidence of the clinical utility of CSF as an alternative liquid biopsy for NSCLC patients with LM.

## Materials and Methods

**Figure 1:**
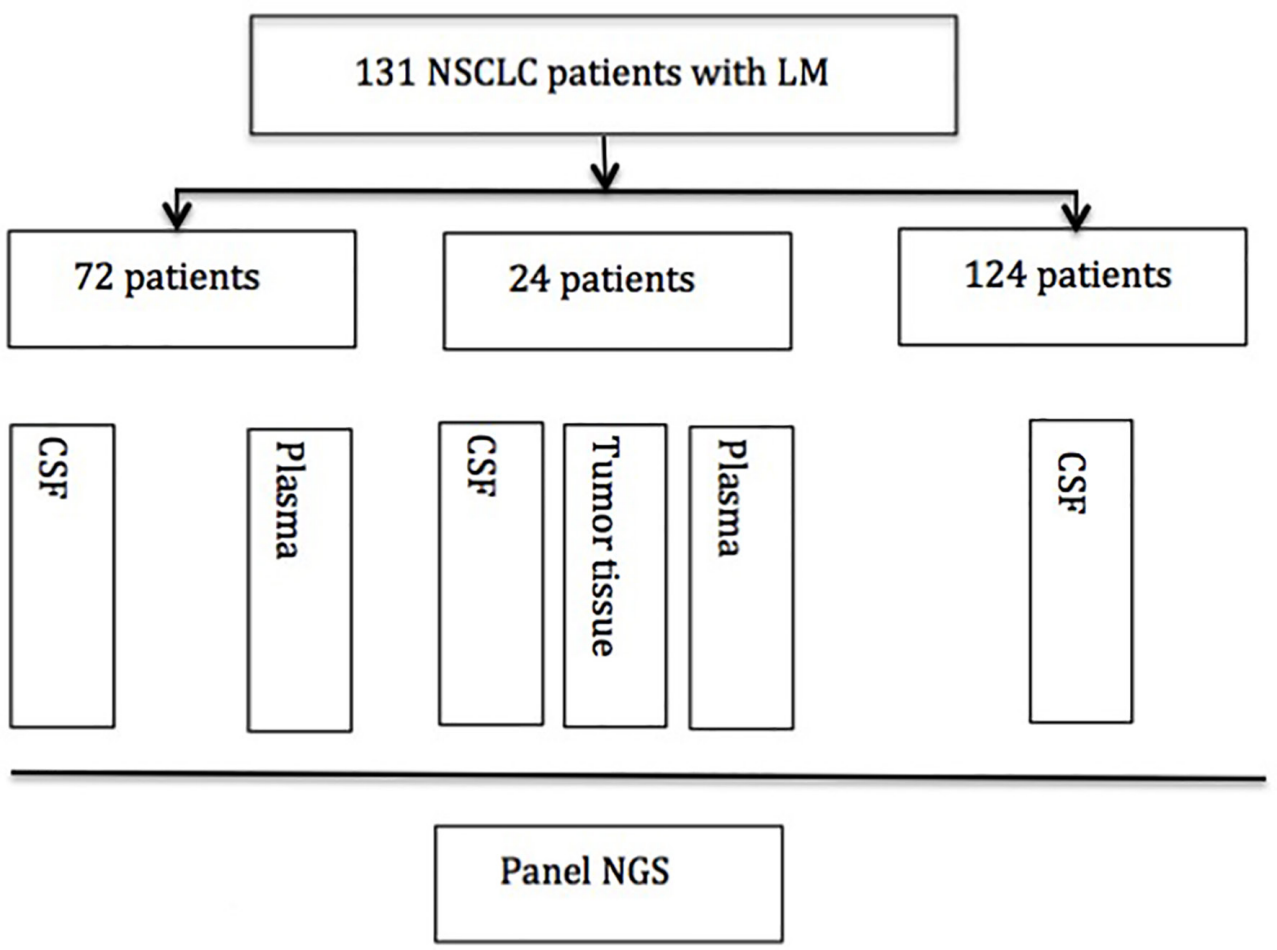
Experimental profile. NSCLC, nonsmall cell lung cancer; CSF, cerebrospinal fluid; NGS, next-generation sequence.

An overview of the design of the study is shown in **Figure 1**.

### Patients

One hundred thirty-one NSCLC patients with suspected LM who underwent lumbar puncture between February 2015 and February 2018 in Nanjing Drum Tower Hospital and other medical centers were enrolled in this study. The baseline patients’ characteristics have been described in number (percentage). Seventy-two matched plasma samples and 24 matched tumor tissue samples were also collected in this study. Briefly, approximately 10 ml of CSF was collected *via* lumbar puncture for cytology examination and next-generation sequencing (NGS), and meanwhile, 10 ml of plasma was collected for NGS. Available primary lung tumor tissues were obtained from the formalin-fixed paraffin-embedded (FFPE) specimen of CT-guided biopsy or biopsy through bronchoscopy. The diagnosis criteria of LM were based on imaging of brain MRI and CSF cytology. MRI findings suggest LM are the pathological enhancement of the leptomeninges of the brain, cranial nerves, and spinal cord. Some patients had no MRI findings, but symptoms indicated LM and CSF cytology confirmed the diagnosis of LM. CSF cytology was reviewed by two independent and experienced pathologists. All patients provided signed informed consent, and the study protocol was approved by the Research Ethics Committee of Nanjing Drum Tower Hospital.

### DNA Extraction

According to the manufacturer’s protocol, a minimum of 20% tumor content was required for FFPE specimens, from which genomic DNA was isolated using GeneRead DNA FFPE Kit (Qiagen, USA). Cell-free DNA from fresh CSF and plasma were extracted by QIAamp Circulating Nucleic Acid Kit (Qiagen, USA) according to the manufacture’s instructions. Quantification of genomic DNA from FFPE, CSF, and plasma samples were analyzed using Qubit 3.0 with dsDNA HS Assay (Life Technologies, Carlsbad, CA, USA).

### Library and Next-Generation Panel Sequencing

Panel sequencing libraries were prepared using KAPA Hyper Prep Kit (KAPA Biosystems, London, UK) according to the manufacturer’s protocol with modifications for each case (21). In brief, DNA was fragmented and subjected to the process of end-repairing, A-tailing, indexed-adapter ligation, size selection, and PCR amplification. In the step of targeted enrichment, indexed DNA libraries were pooled together for hybridization with customized xGen lockdown probes (Integrated DNA Technologies, Coralville, IA, USA) for predefined 416 tumor-associated gene alterations. Then, enriched libraries were amplified and subjected for next-generation sequencing on the platform of Illumina Hiseq4000 (Illumina, San Diego, CA, USA) with a mean depth of 500× for FFPE samples and 3,000× for ctDNA samples from CSF or plasma.

### Bioinformatics Data Analysis

We used Trimmomatic for raw FASTQ file quality control (below 15 or N bases were removed) (22). Next, reads were mapped to the reference human genome (hg19) using BWA-mem, version 0.7.12 (https://github.com/lh3/bwa/tree/master/bwakit) (23). Local realignment around the indels and base quality score recalibration was performed using the Genome Analysis Toolkit GATK 3.4.0 (https://software.broadinstitute.org/gatk/) (24). The somatic mutation was detected with VarScan2. Meanwhile, somatic variant calls with at least 0.2% mutant allele frequency (MAF) and with at least three supporting reads from both directions were retained. Common SNPs were filtered out by dbSNP (v137) with the 1,000 Genomes database, followed by annotation using ANNOVAR. Genomic fusions were found by FACTERA with default parameters (25). Copy-number variations (CNVs) were identified using ADTEx (http://adtex.sourceforge.net) with default parameters. Somatic CNVs were retained using paired normal/tumor samples for each exon with the cutoff of 0.65 for copy number loss and 1.50 for copy number gain (26).

### Statistical Analysis

We describe baseline patients characteristics using numbers or percentages. We also describe the genetic alterations in different samples using numbers or percentages. The genetic alterations between different groups using the Chi-square test and all analyses were based on R (V.3.4.4).

## Results

### Study Design

We screened and enrolled 131 NSCLC patients with LM for analysis. Among them, 24 patients have matched CSF, plasma, and tissue samples for analysis. Seventy-two patients have matched CSF and plasma samples, and 124 patients have CSF analyzed. The study design was as follows (**Figure 1**).

### Clinical Characteristics of NSCLC Patients With LM

All of the patients were Chinese and diagnosed with nonsmall cell lung cancer. Most patients were diagnosed with lung adenocarcinoma (127/131). Only four were diagnosed with squamous carcinoma. The median age was 53 years old (range, 32–76 years), and among them, 10 patients were less than 40 years old, 69 were between 40 and 64 years old, and 23 were more than 65 years old. Sixty-seven patients were male and 64 were female. The majority of patients (128/131) were diagnosed with stage IV disease, only two were stage III, and one was stage II before LM diagnosis. All baseline characteristics were listed as following (**Table 1**). No patients were diagnosed with LM and brain metastasis simultaneously. The most common symptom was dizziness, and some had a headache. Some other symptoms included nausea, vomiting, changed mental status, and numbness of one limb or both limbs.

**Table 1:** Clinical characteristics of patients.

Characteristics	Cohort [*N* (%)]
Gender	
Female	64 (48.9)
Male	67 (51.1)
Histology at diagnosis	
Adenocarcinoma	127 (97.0)
Squamous	4 (3.0)
Age	
Less than 40	10 (7.6)
40–65	69 (52.7)
More than 65	23 (17.6)
NA	29 (22.1)
Stage at diagnosis	
II	1 (0.8)
III	2 (1.5)
IV	128 (97.7)

NA, not available.

### CSF Exhibited Unique Molecular Features in NSCLC Patients With LM

**Figure 2:**
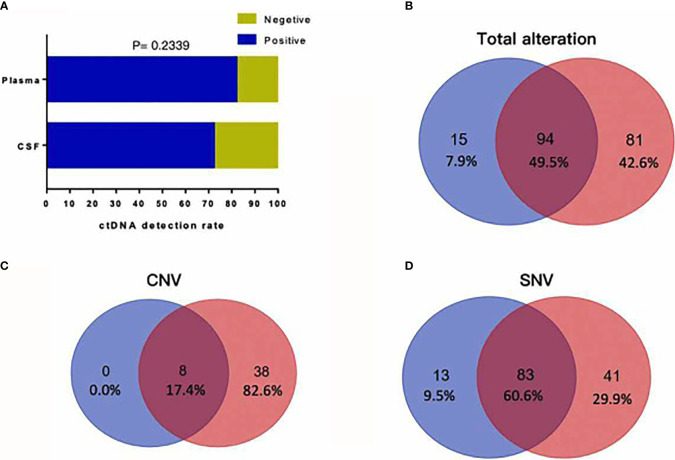
**(A)** cfDNA positive in 52 samples of CSF and in 59 samples of plasma, and the detection rate of cfDNA is not statistically different in CSF and plasma (*p* = 0.2339). **(B)** For detection of genomic alterations, 94 could be detected in both plasma and CSF. However, 81 can be detected in CSF but cannot be found in plasma, while 15 can be found in plasma which cannot be detected in CSF. **(C)** For CNV, eight alterations can be detected both in plasma and CSF and 46 alterations can be found in CSF. **(D)** For SNV, 83 alterations can be found both in CSF and plasma, while 41 can be detected in CSF and 13 in plasma.

**Figure 3:**
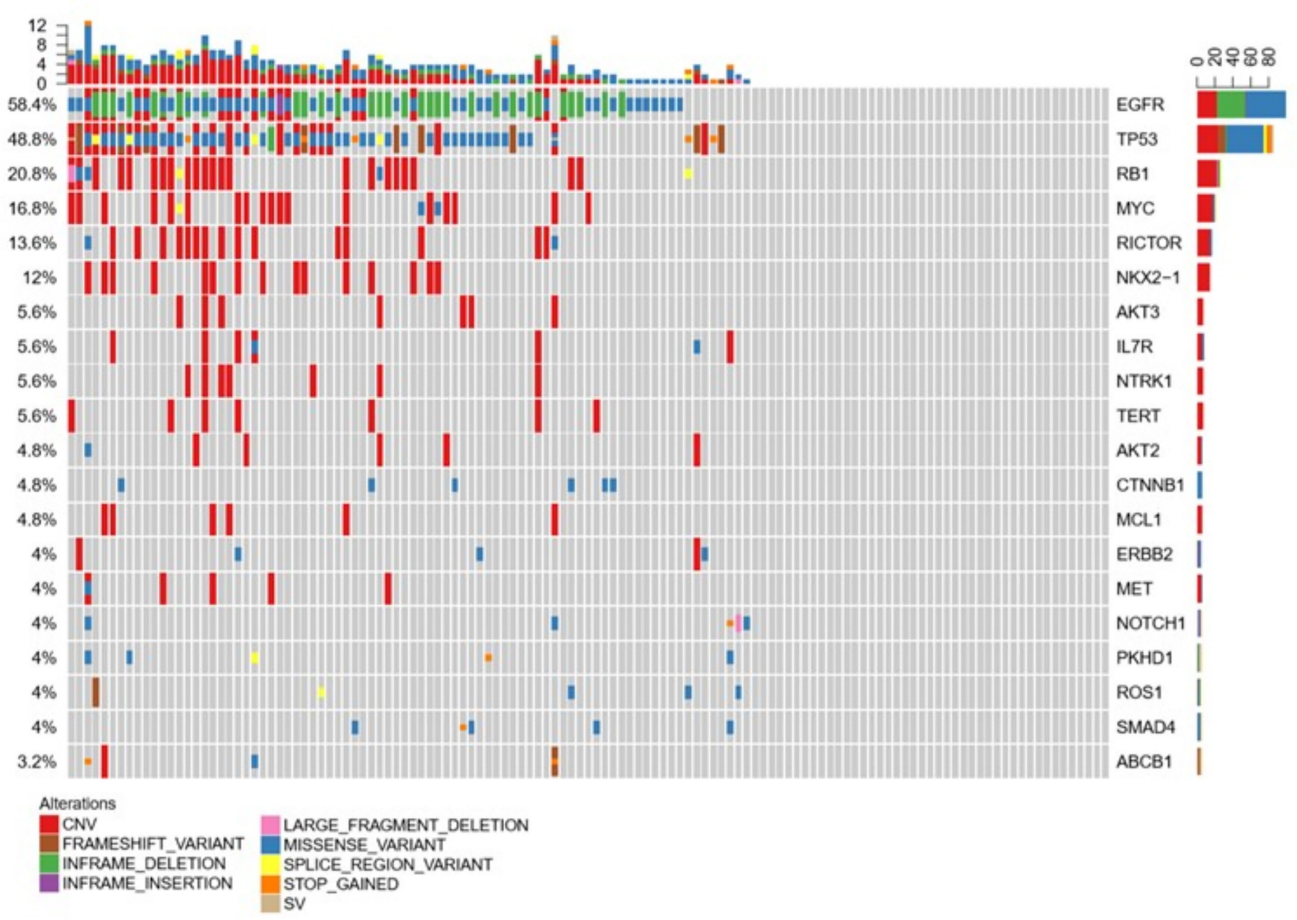
Compared with plasma and tumor tissues, CSF exhibited more CNV alterations and showed unique genomic alterations.

To investigate whether CSF is a better cell-free DNA (cfDNA) method to detect mutations of the tumor as liquid biopsy, we compared 72 samples of CSF and plasma. We found cfDNA positive in 52 samples of CSF and in 59 samples of plasma, and the detection rate of cfDNA is not statistically different in CSF and plasma as **Figure 2A** shows (*p* = 0.2339). **Figure 2B** shows that for the detection of genomic alterations, 94 could be detected in both plasma and CSF. However, 81 can be detected in CSF but cannot be found in plasma, while 15 can be found in plasma which cannot be detected in CSF. For CNV, eight alterations can be detected both in plasma and CSF and 46 can be found in CSF, as shown in **Figure 2C**. For SNV, 83 alterations can be found both in CSF and plasma, while 41 can be detected in CSF and 13 in plasma, as shown in **Figure 2D**. Compared with plasma and tumor tissues, CSF exhibited more CNV alterations and showed unique genomic alterations (**Figures 2** and **3**). The difference of the certain gene alteration profiling identified in our 124 CSF was compared with their alterations in The Cancer Genome Atlas (TCGA) Pan-lung cancer (1,144 samples), the TCGA lung adenocarcinoma (566 samples), and the TCGA lung squamous cell carcinoma (511 samples) (**Supplementary Figures S1–S3**).

### CSF Is Not Inferior to Plasma as Liquid Biopsy

**Figure 4:**
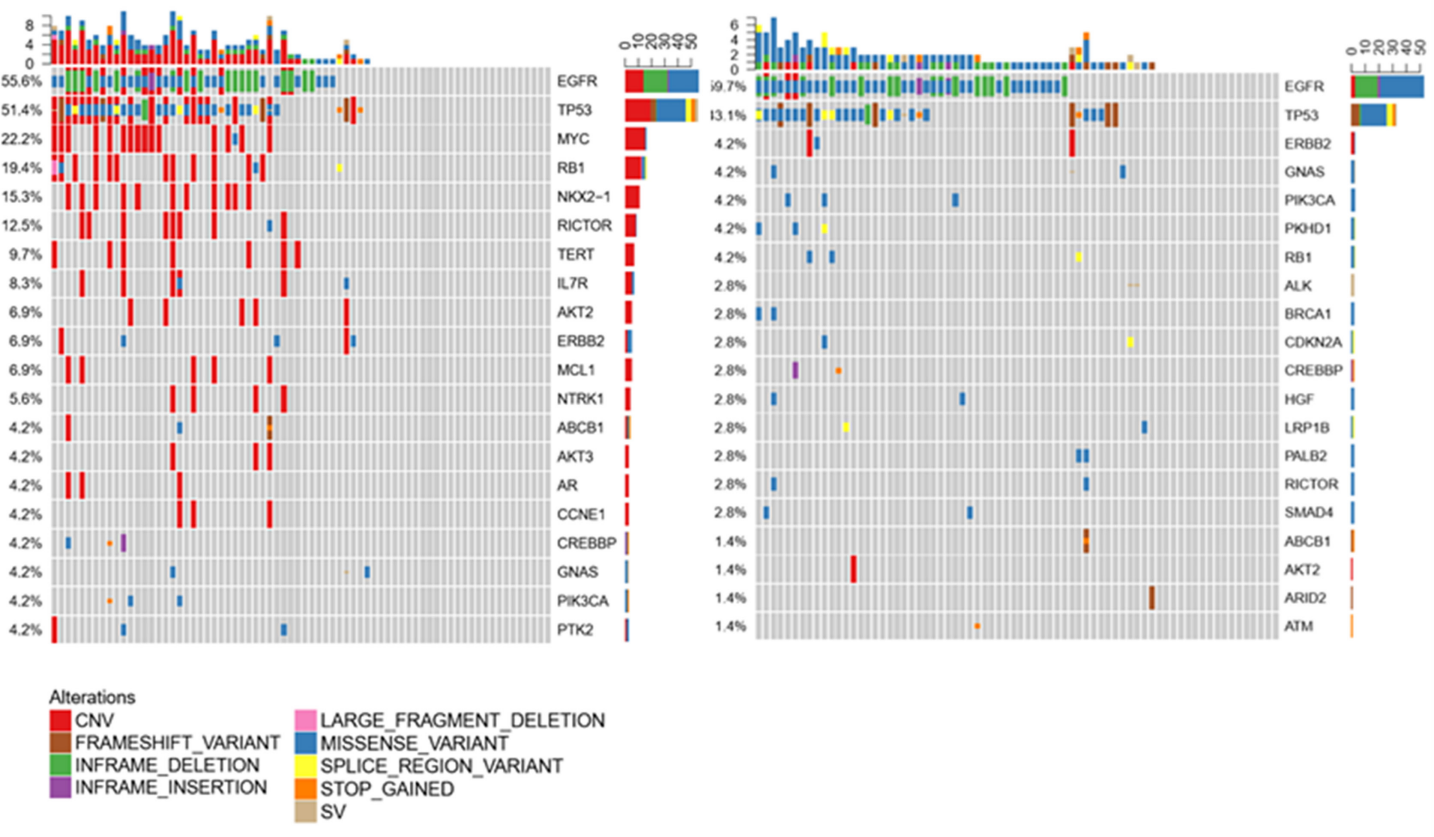
Molecular features in 72 samples were compared in CSF and plasma.

Liquid biopsy is an alternative method to detect mutations for patients if tumor samples are not available. For LM patients, CSF and plasma can be easily obtained. To clarify whether CSF can also be a good method to test the mutations, we compared the CSF and plasma molecular features in 72 samples. Consistent with previous data, EGFR and TP53 were still common both in CSF and plasma, indicating both samples are suitable for mutation testing. However, in CSF, most mutation types were CNV. In plasma, most were missense variants. Except for EGFR and TP53, the most common mutations were MYC, RB1, and NOX2-1 in CSF, but ERBB2, GNAS, and PIK3CA in plasma, as shown in **Figure 4**. These results indicated that CSF molecular features were different from those in plasma. However, for the most important target EGFR, both CSF and plasma can be detected as the most common mutation (**Figure 4**).

### Profiling of Primary Tumor, LM-CSF, and LM-Plasma by Next-Generation Panel Sequencing

**Figure 5:**
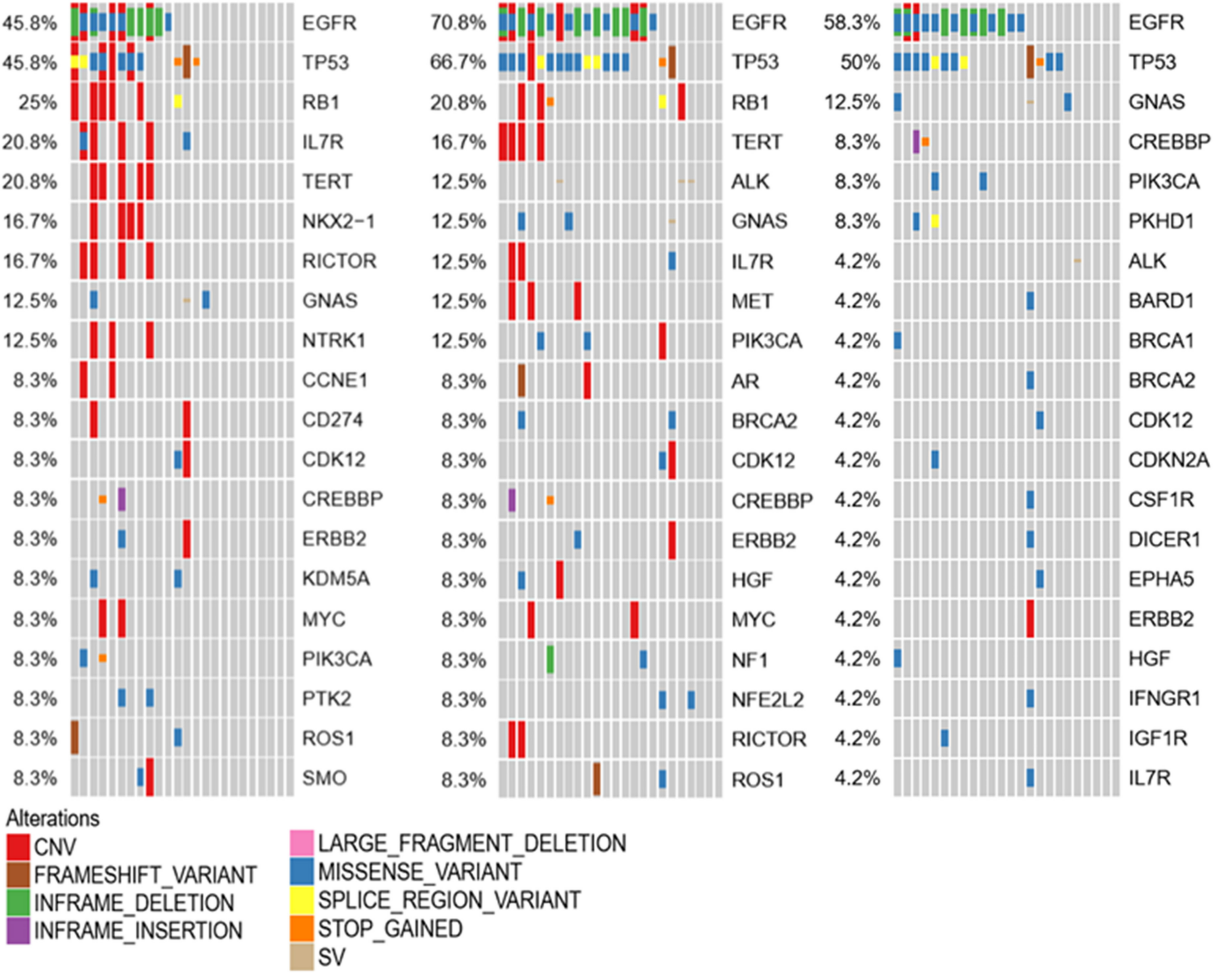
Molecular features in 24 samples were compared between CSF, plasma, and tissue.

To investigate the molecular mechanism of LM, genetic alterations of the primary tumor, LM-CSF, and LM-plasma were compared with each other. The primary tumors were collected before LM diagnosis. LM-CSF and LM-plasma were collected simultaneously and analyzed with the primary tumor. We investigated 24 paired samples of CSF, plasma, and tumor tissues. We found that unique molecular features of CSF compared with tumor tissues and plasma. IL7R, NOX2-1, and NTRK1 were more frequent in CSF but not that frequent both in tumor tissues and plasma. Consistently however, EGFR, TP53, and RB1 were most frequent in CSF, plasma, and tumor tissues, as shown in **Figure 5**.

### EGFR and ALK Detection in CSF

Since EGFR and ALK are the most common and critical actionable targets in NSCLC, we also used panel sequencing to detect EGFR and ALK in CSF. As **Table 2** shows, we found 36 L858 of exon 21 and 32 of 19del and five other subtypes of EGFR mutation. In total, we found 61 samples of EGFR mutation in 124 CSF. The detection rate is almost 50%, consistent with the data from other studies. Interestingly, we demonstrated 11 cases of T790 mutation before any EGFR-TKI treatment, which indicates that these patients may not respond to the first or second generation of EGFR-TKIs. In regard to ALK, three cases were found positive, and the detection rate is low compared with other studies. One reason may be attributed to that panel sequencing is not a preferred method for ALK translocation.

**Table 2:** EGFR/ALK detection in 124 CSF samples.

EGFR/ALK detection			
EGFR	L858	19del	Others
	36	32	5
EGFR+T790M	8	3	
ALK	EML4-ALK	Others	
	3	0	

CSF, cerebrospinal fluid.

### Other Gene Alterations in CSF

We analyzed the CSF samples of 124 patients with LM. The panel sequencing revealed that the most common mutation that occurred was EGFR, which occurred at 58.4% of the CSF samples. This is consistent with previous data because most of the patients are adenocarcinoma and all of the patients are Asians. Interestingly, TP53 and RB1 were also very common in these samples. TP53 occurred in approximately 50% and RB1 in 20.8% of these samples (see **Table 3**). TP53 and RB1 co-occurring expression in tumor tissues in some studies indicated that some tumors might transform to small cell lung cancer (SCLC). We found that co-occurring EGFR/TP53 was not different in CSF, plasma, or in tumor tissue. However, co-occurring EGFR/RB1 and triple-positive EGFR/TP53/RB1 were high in CSF, compared with plasma or tumor tissues (*p* = 0.005 for EGFR/TP53 and *p* = 0.0069 for EGFR/TP53/RB1, respectively). Other gene alterations included NTRK1, MET Proto-Oncogene, and ROS1, and we listed the uncommon driver genes, as show in **Table 4** and **Figure 2**.

**Table 3:** Co-occurring driver genes in CSF/plasma/tumor samples.

Samples	CSF (124%)	Plasma (75%)	Tissue (36%)	*p*-Value
EGFR	73 (58.9)	45 (60)	24 (66.7)	0.72
EGFR+T790M	11 (8.9)	9 (12)	2 (5.6)	0.56
EGFR/TP53	51 (41.1)	24 (32.0)	19 (52.8)	0.10
EGFR/RB1	25 (20.2)	2 (2.7)	3 (8.3)	0.0005
EGFR/TP53/RB1	20 (16.1)	2 (2.7)	3 (8.3)	0.0069

CSF, cerebrospinal fluid.

**Table 4:** Other actionable driver genes in 124 CSF samples.

Samples	CSF (124)	Plasma (75)	Tumor tissue (36)
Other driver genes	10 (8.1%)	3 (4%)	4 (11.1%)
ERBB2 amplification	2	0	2
ERBB2 G668R	1	0	0
ERBB2 Q711H	1	0	0
ERBB2 N571S	1	0	0
BRAF V600E	1	1	0
MET amplification	5	0	3
MET D864H	1	0	0
ERBB2 Q799H	0	0	1
RET fusion (RET-CCDC6)	0	1	0

CSF, cerebrospinal fluid.

## Discussion

NSCLC patients with LM have increased significantly as the survival of NSCLC patients is extended. Some studies suggested that liquid biopsy of CSF is more sensitive than liquid biopsy of plasma to detect targetable alterations (27). In our study, we collected and sequenced more than 130 CSF samples of NSCLC patients with LM. We first demonstrated that CSF is concordant with tumor and plasma to detect drive genes for NSCLC. Secondly, we showed the unique molecular feature of CSF in NSCLC patients with LM, indicating that screening of CSF is a suitable liquid biopsy for NSCLC patients with LM. Thirdly, we have found more pretreatment T790M in NSCLC patients with LM. We assumed that it is important because the EGFR T790M subgroup is not responding to the first or second generation of EGFR-tyrosine kinase inhibitor therapy.

Targeted therapy has dramatically changed treatment approaches for NSCLC (28). Targetable mutation screening has been recommended as a standard of care (29). However, tumor tissue often finds it difficult to obtain or yield unsatisfactory DNA quantity or quality. Liquid biopsy use cell-free DNA shed from the tumor in circulation as a surrogate for tumor tissues. With technology developments, the sensitivity and specificity of cfDNA detection assays have improved significantly. Plasma is the most common material for liquid biopsy and has been researched intensively in clinical practices (30). However, for NSCLC patients with LM, CSF may represent a more comprehensive tool to analyze actionable mutations or to monitor the resistance mechanisms of targeted therapy (31). In our study, we found approximately 50% of EGFR mutations in CSF samples, including eight uncommon mutations and three ALK arrangements. This result is consistent with the previous data and also indicated that panel sequence is suitable for testing gene arrangement in liquid. Tumor tissue heterogeneity can be explained by genetic heterogeneity (intra- and intertumor heterogeneities). However, liquid biopsy can capture tumor heterogeneity more efficiently than is allowed by tissue biopsy.

Except for EGFR and ALK, we also found other interesting, unique gene features in CSF samples. NTRK1 copy number variation but not fusions were found in 5.6% of CSF samples. MET and ROS1 have also occurred in CSF samples of NSCLC with LM. We, however, showed that most of the variants are copy number variants, not fusions or rearrangements that have been shown targetable in NSCLC. Therefore, the significance of CNV of these genes has to be explored in the future.

Co-occurring genetic alterations have been extensively studied in EGFR-mutant NSCLC (32). Recent studies have found that baseline TP53 mutation is significantly associated with shorter survival in EGFR mutant patients treated with EGFR-TKI (33). Co-occurring genetic alterations in the plasma of patients, including WNT/b-catenin and cell-cycle-gene alterations, limited EGFR TKI response in EGFR mutant NSCLC (34). In our study, EGFR is the predominant mutation in CSF, plasma, and tumor tissues. TP53 is the second co-occurring expression of EGFR and TP53 in 51 cases, indicating that these patients may have inferior survival.

The *RB1* gene is the first tumor suppressor gene found as the cause of retinoblastoma (35). Loss of *RB1* was found in 100% of sequences of SCLC tumors in humans, and RB1 mutation is strikingly more frequent in NSCLC with SCLC transformation (SCLC-T) than that remains in NSCLC (33). However, NSCLC patients harboring RB1 mutations do not necessarily transform to SCLC (36). In EGFR mutant lung cancer, one study found that the most frequent accompanying mutations were TP53 and RB1, and these accompanying mutations were associated with poor survival (37). We found that the most frequent accompanying gene alterations were TP53 and RB1 in CSF samples and tumor tissues. However, in plasma of matched tumor tissues, plasma, and CSF samples, we found that the most frequent accompanying alterations were TP53 and GNAS in plasma. These findings indicated that CSF samples could be more prognostic than plasma.

Another study demonstrated that triple-positive EGFR/TP53/RB1 constitute approximately 5% of all EGFR mutant lung adenocarcinoma, and these co-occurring mutations are uniquely associated with high-risk SCLC transformation (seven of 39, 18%) and poor survival (38). In our study, triple alterations has been found in 20 cases in CSF, but whether it is associated with poor survival or high risk of SCLC transformation needs to be further analyzed with long-time follow-up.

This study has several limitations. First, not all samples are paired with tumor tissues and plasma samples, which cautions the interpretation of these results. Second, the CSF and plasma samples are not large enough. Third, there was no survival time of patients in this study, which means that whether certain gene alterations can impact survival is unclear.

## Conclusion

CSF samples demonstrate unique molecular features and are concordant with tumor tissues regarding the molecular analysis. Thus, it may represent a better surrogate for NSCLC patients for molecular analysis when no tumor tissues are available.

## Data Availability Statement

According to the national legislation/guidelines, specifically the Administrative Regulations of the People’s Republic of China on Human Genetic Resources (http://www.gov.cn/zhengce/content/2019-06/10/content_5398829.htm, http://english.www.gov.cn/policies/latest_releases/2019/06/10/content_281476708945462.htm), no additional raw data is available at this time. Data of this project can be accessed after an approval application to the China National Genebank (CNGB, https://db.cngb.org/cnsa/). Please refer to https://db.cngb.org/ or email: CNGBdb@cngb.org for detailed application guidance. The submission ID (sub021661) and project accession (CNP0001938) should be included in the application.

## Ethics Statement

This research was approved by the Nanjing Drum Tower Hospital Affiliated to Medical School of Nanjing University Institutional Review Board. The sample was provided upon written informed consent. Written informed consent for participation was not required for this study in accordance with the national legislation and the institutional requirements.

## Author Contributions

YW, FJ, RX, ML, CY, GZ, HC, LM, and HX conceived the study and its design. YL, HL, QZ, MS, GZ, and YY helped in the sample collection. YW, YS, and HX performed experiments and data analyses. YW and HX drafted and revised the manuscript. All authors contributed to the article and approved the submitted version.

## Funding

This study was supported by grants from the Postdoctoral Funding of Nanjing Drum Tower Hospital Affiliated to Medical School of Nanjing University; The Recruitment Program of Overseas High-Level Young Talents, “Innovative and Entrepreneurial Team” (No. (2018) 2015), Science and Technology Grant (BE2019758), and the Six Talent Peaks Project (TD-SWYY-007) of Jiangsu Province and High-Level Talents Program of Nanjing Medical University; Xisike Clinical Oncology Research Foundation (CSCO-Haosen, Y-HS202102-0177); and The Medical Interdisciplinary Research Funding of Henan University (No. CJ1205A0240011).

## Conflict of Interest

Author YS was employed by Nanjing Geneseeq Technology Inc.

The authors declare that the research was conducted in the absence of any commercial or financial relationships that could be construed as a potential conflict of interest.

## Publisher’s Note

All claims expressed in this article are solely those of the authors and do not necessarily represent those of their affiliated organizations, or those of the publisher, the editors and the reviewers. Any product that may be evaluated in this article, or claim that may be made by its manufacturer, is not guaranteed or endorsed by the publisher.
