# Supplementary material for: Unique Genomic Alterations of Cerebrospinal Fluid Cell-Free DNA Are Critical for Targeted Therapy of Non-Small Cell Lung Cancer With Leptomeningeal Metastasis
Source: Front Oncol. 2021 Oct 4;11:701171. doi: 10.3389/fonc.2021.701171 (PMC8522975; doi:10.3389/fonc.2021.701171)
Supplement: Supplementary file 1 [file DataSheet_1.pdf]

Supplementary data for

Unique genomic alterations of cerebrospinal fluid cell-free DNA are critical for targeted therapy of non-small cell lung cancer with leptomeningeal metastasis

Figure S1

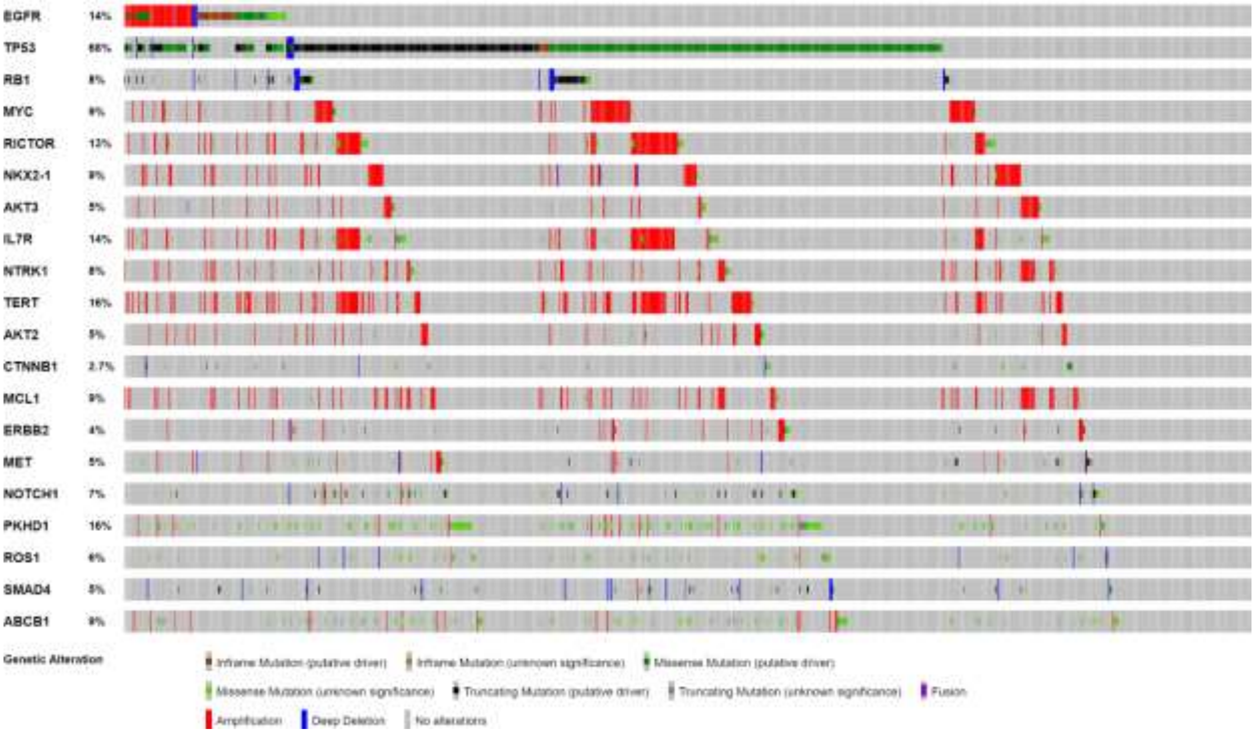

Figure S1 The certain gene alterations profiling identified in our 124 CSF was compared with their alterations in the TCGA Pan-lung cancer (1144 samples).

Figure S2

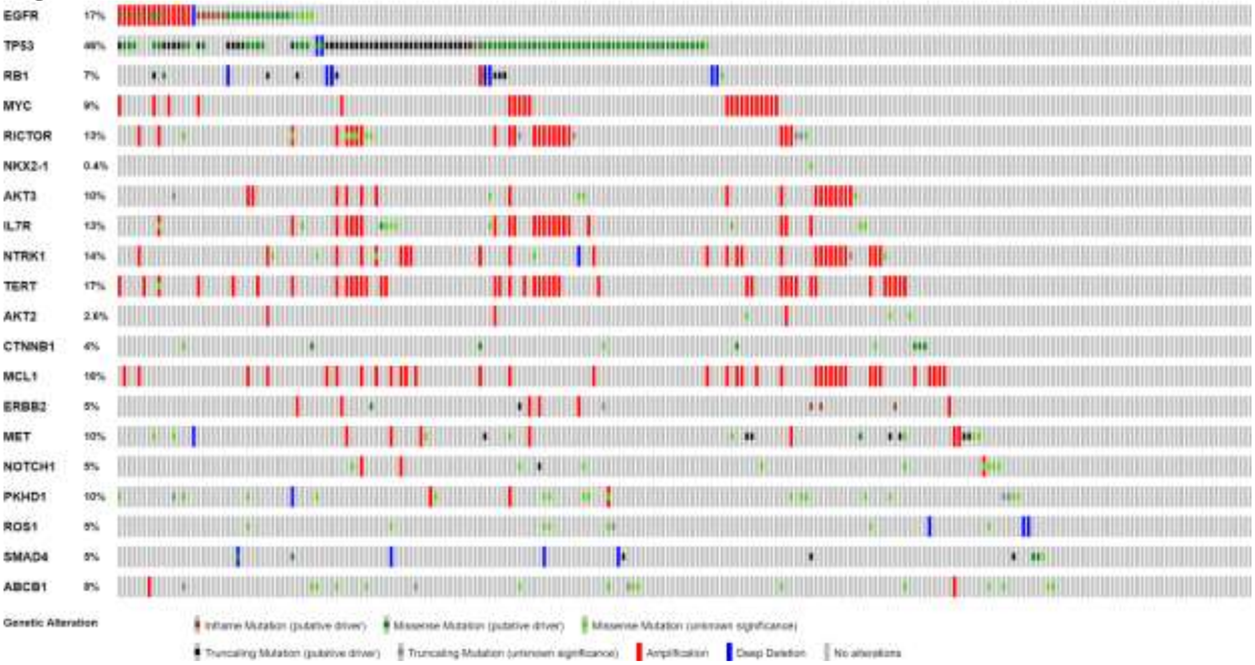

Figure S2 The certain gene alterations profiling identified in our 124 CSF was compared with their alterations in the TCGA lung adenocarcinoma (566 samples).

Figure S3

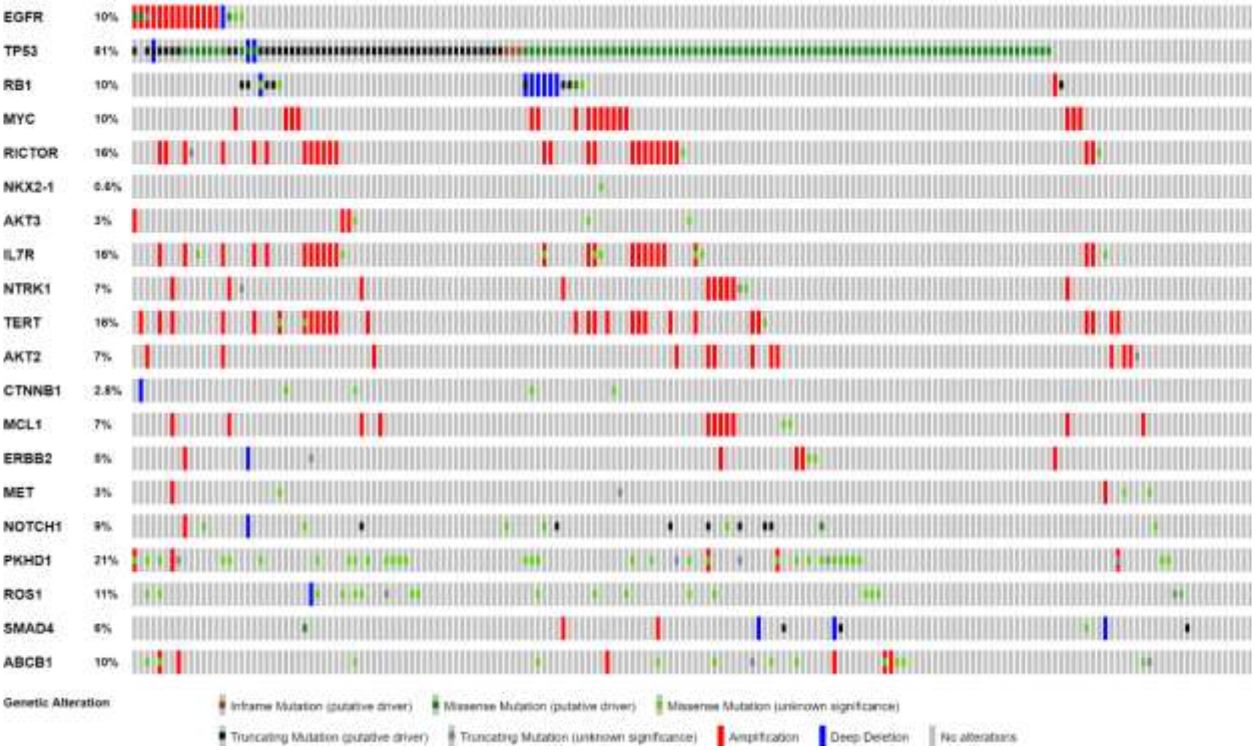

Figure S3 The certain gene alterations profiling identified in our 124 CSF was compared with their alterations in the TCGA lung squamous cell carcinoma (511 samples).
